# Estimation of Transmission Parameters of H5N1 Avian Influenza Virus in Chickens

**DOI:** 10.1371/journal.ppat.1000281

**Published:** 2009-01-30

**Authors:** Annemarie Bouma, Ivo Claassen, Ketut Natih, Don Klinkenberg, Christl A. Donnelly, Guus Koch, Michiel van Boven

**Affiliations:** 1 Faculty of Veterinary Medicine, Utrecht University, The Netherlands; 2 Central Veterinary Institute, Wageningen University and Research Centre, The Netherlands; 3 National Veterinary Drug Assay Laboratory, Bogor, Indonesia; 4 MRC Centre for Outbreak Analysis and Modelling, Department of Infectious Disease Epidemiology, Imperial College London, London, United Kingdom; 5 Centre for Infectious Disease Control, National Institute for Public Health and the Environment, The Netherlands; Erasmus Medical Center, The Netherlands

## Abstract

Despite considerable research efforts, little is yet known about key epidemiological parameters of H5N1 highly pathogenic influenza viruses in their avian hosts. Here we show how these parameters can be estimated using a limited number of birds in experimental transmission studies. Our quantitative estimates, based on Bayesian methods of inference, reveal that (i) the period of latency of H5N1 influenza virus in unvaccinated chickens is short (mean: 0.24 days; 95% credible interval: 0.099–0.48 days); (ii) the infectious period of H5N1 virus in unvaccinated chickens is approximately 2 days (mean: 2.1 days; 95%CI: 1.8–2.3 days); (iii) the reproduction number of H5N1 virus in unvaccinated chickens need not be high (mean: 1.6; 95%CI: 0.90–2.5), although the virus is expected to spread rapidly because it has a short generation interval in unvaccinated chickens (mean: 1.3 days; 95%CI: 1.0–1.5 days); and (iv) vaccination with genetically and antigenically distant H5N2 vaccines can effectively halt transmission. Simulations based on the estimated parameters indicate that herd immunity may be obtained if at least 80% of chickens in a flock are vaccinated. We discuss the implications for the control of H5N1 avian influenza virus in areas where it is endemic.

## Author Summary

Outbreaks of highly pathogenic H5N1 avian influenza in poultry first occurred in China in 1996. Since that time, the virus has become endemic in Asia, and has been the cause of outbreaks in Africa and Europe. Although many aspects of H5N1 virus biology have been studied in detail, surprisingly little is known about the key epidemiological parameters of the virus in its avian hosts (the length of time from infection until a bird becomes infectious, the duration of infectiousness, how many birds each infectious bird will infect). In this paper we show, using experimental transmission studies with unvaccinated and vaccinated chickens, that H5N1 avian influenza induces a short duration of infectiousness (∼2 days) and a very short period of time from infection until infectiousness (∼0.25 day) in unvaccinated chickens. Furthermore, while transmission was efficient among unvaccinated birds, no bird-to-bird transmission was observed in vaccinated chickens. Our results indicate that it may be difficult to curb outbreaks by vaccination after an introduction in a flock has been detected. On the other hand, preventive vaccination could be effective in preventing virus introductions and limiting the size of outbreaks.

## Introduction

Highly pathogenic avian influenza virus strains of the H5 or H7 subtypes are noted for being highly contagious among various bird species and inducing high mortality rates in poultry. Although outbreaks of highly pathogenic avian influenza have been reported since the 1950s the current focus is on the H5N1 subtype. The first outbreaks of H5N1 were reported in Hong Kong in 1997 [1]–[2]. Since then the virus has spread to South East Asia, Africa, and Europe. The outbreaks in Europe were controlled by rapid depopulation of infected premises, pre-emptive culling of neighbouring farms, movement restrictions, and zoo-sanitary measures [3]–[5]. In Asia, however, the disease has become endemic, and control by means of culling in conjunction with movement restrictions and zoo-sanitary measures is both infeasible socio-economically and unlikely to result in elimination [6]–[10]. Therefore, vaccination is the most widely used containment strategy. For instance, in Indonesia alone more than 400 million of vaccine doses have been administered since 2004.

Despite the fact that aspects of H5N1 avian influenza biology have been studied in detail, ranging from molecular studies of host range factors, phylogenetic analyses aimed at unravelling the virus' evolutionary pathways, surveillance of H5N1 in wild birds, studies into the clinical course of H5N1 infections in humans, and vaccine efficacy and safety studies, there is scant information of the basic epidemiological characteristics of H5N1 viruses in their avian hosts. Specifically, little is known about the infectious period of H5N1 in various host species, the duration of the latent period, and the transmissibility of the virus from bird to bird. For a proper understanding of the transmission dynamics of the virus and to be able to assess the potential impact of control measures such as vaccination, however, this information is crucial. For instance, it is well-known that both the invasion prospects of the virus as well as the number of individuals ultimately infected are critically affected by the (distribution of the) infectious period and transmission parameter. The (distribution of the) period of latency is also of importance since it is a key factor affecting the initial growth rate and duration of an epidemic [11]–[13].

Here we present and analyze experimental transmission studies with highly pathogenic H5N1 avian influenza virus (A/Chicken/Legok/2003) in chickens to obtain quantitative estimates of key epidemiological parameters. Specifically, we performed experiments in which an artificially infected chicken was placed in a cage with a susceptible contact bird, and in which the transmission chain was monitored by taking daily samples from the trachea and cloaca [14]–[16]. The samples were subsequently tested for the presence of virus by egg-culture. In addition, blood samples were taken weekly to determine the antibody response to infection. In all, two experiments, each containing 11 trials, were carried out with unvaccinated chickens, two experiments of 11 trials were performed using an H5N1 inactivated oil emulsion vaccine which contains a strain that is identical to the challenge virus (A/Chicken/Legok/2003), and four experiments of 11 trials were carried out with two heterologous H5N2 inactivated oil emulsion vaccines (A/Turkey/England/N28/73 and A/Chicken/Mexico/232/94/CPA) that are both genetically and antigenically distant from the challenge virus.

The experiments are analyzed by tailored statistical methods based on a SEIR (susceptible-exposed-infectious-removed) epidemiological model. In this way all estimated parameters have a clear-cut biological interpretation (mean and variance of the latent and infectious period, transmission rate, reproduction number). Here we use two different methods of analysis. The first uses final size data i.e. the number of birds that are ultimately infected, and is aimed at estimation of the reproduction number [17]. The second approach uses all available information and is based on Bayesian inference that relies on Markov Chain Monte Carlo (MCMC) techniques [18]–[21]. This allows one to estimate not only the reproduction number, but also other epidemiological parameters of interest. The main advantage of our controlled experimental setup over field studies [6],[7],[22] is that the parameters of interest can be estimated with high precision using a limited number of birds. In addition, our controlled experimental setup makes it possible to ascribe differences between control and treatment groups directly to the treatment without having to take into account the potential effect of confounding variables (e.g., age and size of the birds, stocking density, feeding status).

## Results

### Infection and disease

All inoculated unvaccinated birds (Tables 1 and 2) showed signs of infection (depression, labored breathing), shed virus from both the trachea and cloaca (apart from a single bird in Table 2), and died within a few days after infection (range: day 2–day 3). Furthermore, all contact birds died on day 4 or on day 5 after infection of the inoculated bird, indicating rapid infection as well as rapid progression of the disease towards death. In the experiments with unvaccinated birds 8 out of 22 birds escaped infection. These birds did not show signs of disease, did not shed detectable virus, and remained serologically negative when tested in the HI assay at days 7 and 14.

**Table 1:** Overview of the transmission experiment with unvaccinated birds (low infection dose)*.

bird type	days post challenge							
	0	1	2	3	4	5	6	7
i	−/−	+/+	+/+	†				
c	−/−	−/−	−/−	+/+	†			
i	−/−	+/+	+/+	†				
c	−/−	−/−	+/−	+/+	†			
i	−/−	+/+	+/+	†				
c	−/−	+/+	−/−	+/+	−/+	†		
i	−/−	+/+	+/+	†				
c	−/−	−/−	−/−	−/−	−/−	−/−	−/−	−/−#
i	−/−	+/+	+/+	†				
c	−/−	−/−	+/−	+/+	+/+	†		
i	−/−	+/−	+/+	†				
c	−/−	−/−	−/−	−/−	−/−	−/−	−/−	−/−#
i	−/−	+/+	+/+	†				
c	−/−	−/−	+/−	+/+	+/+	†		
i	−/−	+/+	+/+	†				
c	−/−	−/−	−/−	+/+	+/+	†		
i	−/−	+/+	+/+	†				
c	−/−	−/−	**+/**−	**+/+**	**+/+**	†		
i	−/−	+/+	+/+	†				
c	−/−	−/−	+/−	+/+	†			
i	−/−	+/+	+/+	†				
c	−/−	−/−	−/−	+/+	†			

*: the infection dose is 0.2 ml of 10^5^ EID_50_ intranasally (0.1 ml) and intraocularly (0.1 ml).

i: inoculated bird; c: contact bird; x/y: test result for virus isolation in the trachea/cloaca.

**†:** bird died.

# the bird was alive at the end of the experiment and tested negative in the serological test.

**Table 2:** Overview of the transmission experiment with unvaccinated birds (high infection dose)*.

bird type	days post challenge							
	0	1	2	3	4	5	6	7
I	−/−	+/+	†					
C	−/−	−/−	−/−	−/−	†			
i	−/−	−/+	†					
c	−/−	−/−	−/−					#
i	−/−	+/+	†					
c	−/−	−/−	−/−					#
i	−/−	−/+	†					
c	−/−	−/−	−/−					#
i	−/−	+/+	†					
c	−/−	−/−	−/−					#
i	−/−	+/+	†					
c	−/−	−/−	−/−					#
i	−/−	+/+	†					
c	−/−	−/−	−/−					#
i	−/−	+/+	†					
c	−/−	−/−	−/−	+/+	†			
i	−/−	+/+	+/−	†				
c	−/−	−/−	−/−	−/−	−/+	†		
i	−/−	+/+	†					
c	−/−	−/−	−/−	+/+	†			
i	−/−	+/+	−/−	†				
c	−/−	−/−	+/−	+/+	†			

*: the infection dose is 0.2 ml of 10^6^ EID_50_ intranasally (0.1 ml) and intraocularly (0.1 ml).

i: inoculated bird; c: contact bird; x/y: test result for virus isolation in the trachea/cloaca.

**†:** the bird died.

# the bird was alive at the end of the experiment and tested negative in the serological test.

In the experiments with vaccinated birds no contact birds were infected and only a few of the inoculated birds shed virus on just a few days. In fact, only 7 out of 66 inoculated birds shed virus for a total of 12 days. Of these, virus was isolated from the trachea only on 11 days and from the trachea and cloaca on a single day. None of the vaccinated birds died in the course of the experiments, and no signs of disease were observed in any of the vaccinated birds. Details of the vaccination experiments are given in Tables S2, S3, S4, S5, S6, and S7.

### Transmission in unvaccinated versus vaccinated birds

With regard to transmission, the final size analyses indicate that there are significant differences between the experiments with unvaccinated and vaccinated birds. Table 3 summarizes the results. For the low-dose experiment with unvaccinated birds estimates of the reproduction number are 9.0 (95% confidence interval (CI): 1.9–86) in case of an exponentially distributed infectious period and 3.4 (95%CI: 1.3–7.6) in case of a fixed infectious period. For the high-dose experiment the estimates of the reproduction number are 1.7 (95%CI: 0.40–6.6) in case of an exponentially distributed infectious period and 1.2 (95%CI: 0.37–2.9) in case of a fixed infectious period. Although the difference between the two experiments is not statistically significant (p = 0.23) [23], it does hint at the possibility of a role of the inoculation dose in impacting on the transmission dynamics. If all experiments with unvaccinated birds are combined the outcome is that 14 out of 22 initially susceptible contact birds are infected. In this case the estimates of the reproduction number are 3.5 (95%CI: 1.4–9.6) and 2.0 (95%CI: 1.0–3.5), assuming exponentially distributed and fixed infectious periods respectively, indicating that the virus is able to spread epidemically in unvaccinated populations.

**Table 3:** Overview of the final size analyses.

experiment	final size	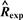 *	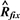 *	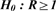 #
low dose (N = 11)	9	9 (1.9–86)	3.4 (1.3–7.6)	1.0/1.0
high dose (N = 11)	5	1.7 (0.40–6.6)	1.2 (0.37–2.9)	0.88/0.77
vaccination^x^ (N = 11)	0	0 (0–0.80)	0 (0–0.67)	0.011/0.0041

*: maximum likelihood estimates of the reproduction number with 95% confidence intervals (between brackets) if the infectious period is exponentially distributed (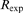) or of fixed duration (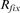).

# p-values of the null hypothesis under the assumption of an exponentially distributed and fixed infectious period.

x all experiments with vaccinated birds (Table S1) yielded a final size of 0. Shown are the results for a single vaccination experiment.

No transmission was observed in all six experiments with vaccinated birds, resulting in a maximum likelihood estimate of the reproduction number of 0. The (two-sided) 95% confidence interval ranges from 0–0.80 or 0–0.67, depending on the assumptions regarding the distribution of the infectious period. Furthermore, the null-hypothesis that the reproduction number is larger than the threshold value 1 can safely be rejected (p = 0.011 in case of an exponentially distributed infectious period, and p = 0.0041 in case of a fixed infectious period). Hence, it is unlikely that an epidemic can occur in vaccinated populations.

### Transmission dynamics in unvaccinated birds

**Figure 1:**
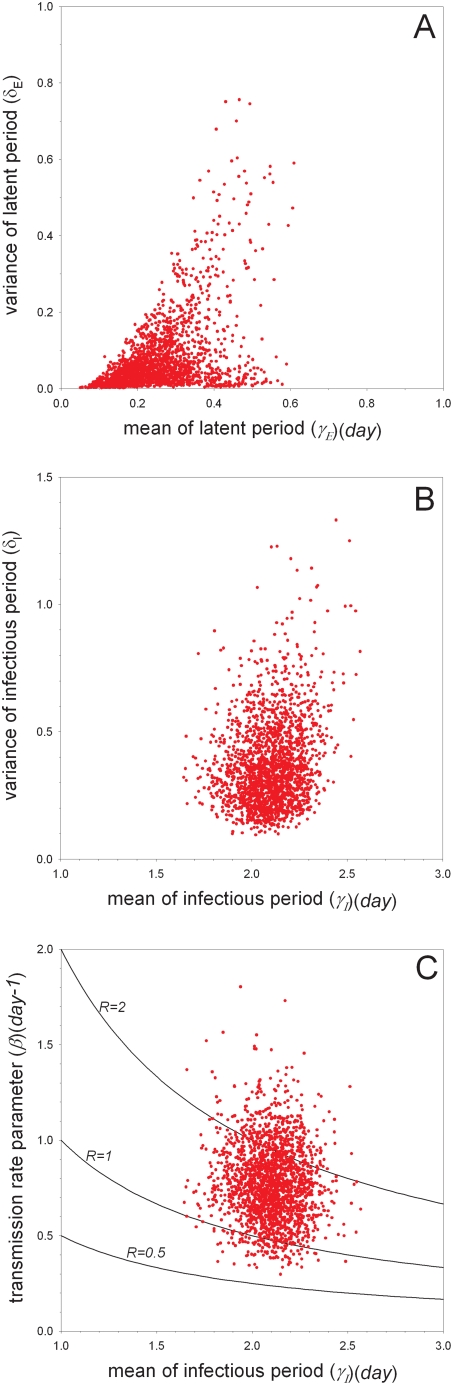
Bayesian analysis of the experiments with unvaccinated birds (scenario B). Shown are samples from the marginal posterior density of the mean versus variance of the latent period (A), the mean versus variance of the infectious period (B), and the mean infectious period versus transmission rate parameter (C). The contours in (C) correspond to specific values of the reproduction number. See Figures S1, S2, and S3 for additional results.

The experiments with unvaccinated birds are analyzed using Bayesian methods to obtain estimates of the transmissibility of the virus and the distributions of the latent and infectious periods. Table 4 and Figures 1–3, S1, S2, S3, S4, and S5 summarize the main findings. In the low-dose experiment (Table 1) as well as the high-dose experiment (Table 2) the estimated mean of the latent period (or, more precisely, the median of the marginal posterior distribution of the parameter determining the mean) is small (0.20 (*day*) or 0.44 (*day*)), as is the variance of the latent period (0.044 or 0.078) (Figures S1 and S2). The estimated means of the transmission parameter are also comparable in the two experiments, ranging from 0.74 (*day^−1^*) in the high-dose experiment to 0.80 (*day^−1^*) in the low-dose experiment. With regard to the infectious period, however, there appear to be differences between the low- and high-dose experiments, with the birds in the low-dose experiment having a substantially longer infectious period. In fact, the estimated mean of the infectious period is 2.5 (*day*) (95%CI: 2.2–2.8 (*day*)) for the low-dose experiment, and 1.3 (*day*) (95%CI: 0.92–1.8 (*day*)) for the high-dose experiment. In both experiments the estimated variance of the infectious period is small (0.16 or 0.13), indicating that the infectious period distribution is narrowly centered around the mean.

**Figure 2 ppat-1000281-g002:**
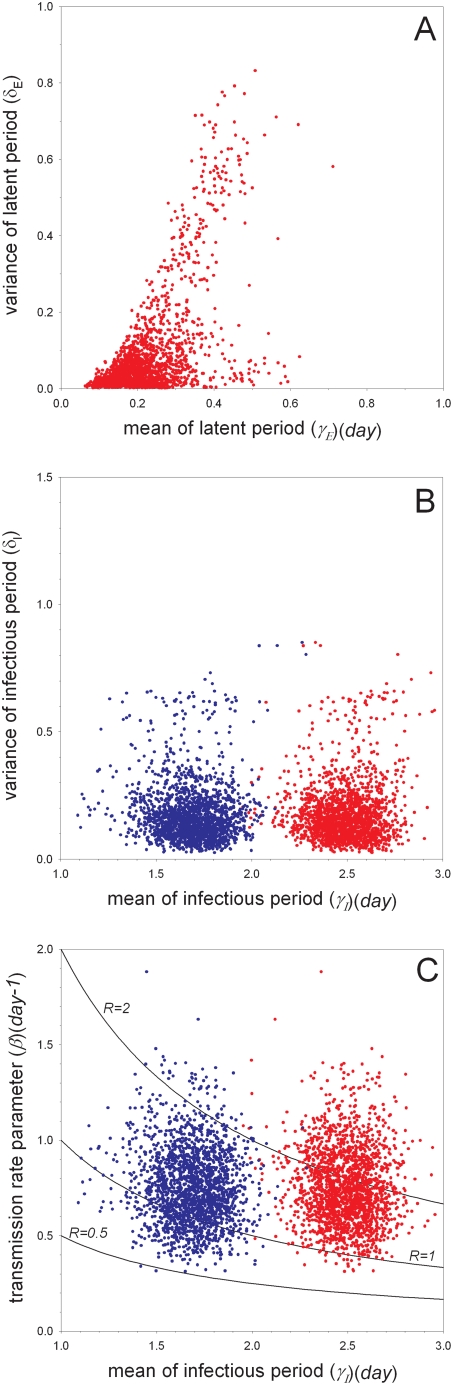
Bayesian analysis of the experiments with unvaccinated birds (scenario C). Shown are samples from the marginal posterior density of the mean versus variance of the latent period (A), the mean versus variance of the infectious period (B), and the mean infectious period versus transmission rate parameter (C). Blue and red dots refer to parameters characterizing the low- and high-dose experiments, respectively. The contours in (C) correspond to specific values of the reproduction number. See Figure S4 for additional results.

**Figure 3:**
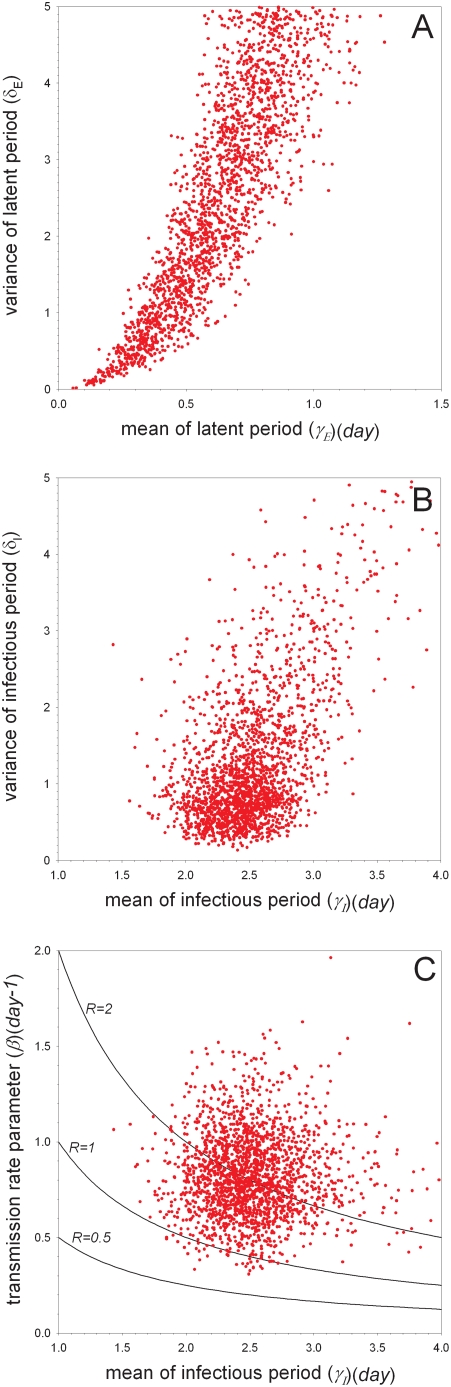
Bayesian analysis of the experiments with unvaccinated birds (scenario D). Shown are samples from the marginal posterior density of the mean versus variance of the latent period (A), the mean versus variance of the infectious period (B), and the mean infectious period versus transmission rate parameter (C). Dots refer to parameters characterizing the contact infections. The contours in (C) correspond to specific values of the reproduction number. See Figure S5 for additional results.

**Table 4:** Overview of the Bayesian analyses.

scenario	description	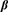 (*day^−1^*)*	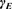 (*day*)*	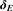 *	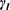 (*day*)*	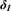 *	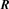 *	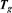 (*day*)*
A1	low-dose experiment	0.80	0.20	0.044	2.5	0.16	2.0	1.2
		(0.38–1.5)	(0.049–0.43)	(0.0020–0.45)	(2.2–2.8)	(0.045–0.48)	(0.96–3.6)	(0.94–1.5)
A2	high-dose experiment	0.74	0.44	0.078	1.3	0.13	0.99	1.8
		(0.27–1.6)	(0.14–0.87)	(0.0026–1.1)	(0.92–1.8)	(0.0097–0.66)	(0.38–2.1)	(1.3–2.3)
B	combined analysis	0.76	0.24	0.043	2.1	0.33	1.6	1.3
		(0.42–1.2)	(0.099–0.48)	(0.0039–0.36)	(1.8–2.3)	(0.15–0.77)	(0.90–2.5)	(1.0–1.5)
C	differences in the mean infectious period	0.73	0.20	0.035	2.5# (2.2–2.8)	0.15	1.8# (1.1–3.0)	1.3
		(0.43–1.2)	(0.094–0.45)	(0.0030–0.54)	1.7# (1.4–2.0)	(0.049–0.58)	1.2# (0.71–2.0)	(1.1–1.5)
D	differences between inoculated/contact birds^x^		0.44	0.038	1.7	0.19		
		0.81	(0.18–0.70)	(0.0011–0.53)	(1.4–2.1)	(0.025–0.58)	2.0	1.2
		(0.44–1.3)	0.62	2.4	2.5	0.96	(1.0–3.5)	(1.2–1.3)
			(0.21–1.0)	(0.26–4.9)	(1.9–3.3)	(0.31–3.7)		

Cells show the median of the marginal posterior distributions with 95% credible intervals (between brackets).

*: 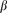: transmission rate parameter; 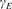: mean of the latent period; 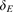: variance of the latent period; 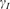: mean of the infectious period; 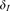: variance of the infectious period; 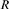: reproduction number; 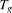: generation interval.

# the upper and lower rows give the parameter estimates for the low and high dose experiments, respectively.

x the upper and lower rows give the parameter estimates for the inoculated and contact birds, respectively. The estimate of the reproduction number is based on the infectious period of the contact infected birds.

We then analyzed the data of the low- and high-dose experiments simultaneously to obtain more precise estimates of the parameters of interest. We considered three scenarios (labeled by B, C, and D) that differ with regard to assumptions on the latent and infectious periods (see Methods). Analysis of the pooled data (scenario B) verifies the earlier indications (scenarios A1–A2) that both the estimated mean and variance of the latent period are small, while the estimated mean of the infectious period (2.1 (*day*)) lies between the estimated means of the infectious period in the analyses of the low- and high-dose experiments (Figure 1). In comparison with the separate analyses of the low- and high-dose experiments the estimated variance of the infectious period increases (0.16 and 0.13 in the low- and high-dose experiments versus 0.33 in scenario B), probably because of the need to accommodate both short (∼1.5 (*day*)) and long (∼2.5 (*day*)) infectious periods. Alternatively, if the infectious period distributions are allowed to differ between the low- and high-dose experiments (scenario C), then the estimated mean infectious periods as well as the corresponding variance estimates revert to values close to those in the separate analyses of the low- and high-dose experiments (Figure 2). Based on Bayes factor (see Methods) the model that allowed for differences in the infectious periods has substantially higher support (BF = 21 for the pair of simulations of competing models with the smallest difference in marginal likelihoods) than the model in which the infectious period distributions in the low- and high-dose experiments are assumed to be equal. Finally, if the latent and infectious period distributions are allowed to differ between inoculated and contact birds (scenario D; Figure 3) there is some evidence that, overall, the infectious period of the contact infected birds was somewhat longer than that of the artificially infected birds (mean 2.5 (*day*)(95%CI: 1.9–3.3) versus mean 1.7 (*day*)(95%CI: 1.4–2.1)). Figure 3 furthermore shows that the variances of the latent and infectious period distributions of the contact infected birds could not be estimated with precision. An extended analysis including alternative informative prior distributions and an artificially extended dataset indicate that this is indeed the case, and that the experiments of Table 1 and 2 do not contain sufficient information to estimate the variance of the latent and infectious periods of the contact infected birds (unless substantial prior information is added)(results not shown).

Two derived epidemiological measures of interest are the reproduction number *R* and the generation interval *T_g_* [11]–[13]. In our setting the reproduction number is given by the product of the infectious period and the transmission rate, while the generation interval is defined as the moment of infection of the contact bird, relative to the time at which the inoculated bird was returned to the cage following inoculation. Overall, the generation interval ranges from an estimated mean of 1.2 (*day*) in scenario A1 and scenario D to 1.8 (*day*) in scenario A2, with limited variation around these estimates. This indicates that the generation interval is short, and lies in the range of 1–2 days. With regard to the reproduction number, we find substantial differences in the reproduction number between the low- and high-dose experiments. In fact, the estimated reproduction number is 2.0 (95%CI: 0.96–3.6) in the low-dose experiment, and 0.99 (95%CI: 0.38–2.1) in the high-dose experiment. This difference can be ascribed to differences in the mean infectious period in the low- versus high-dose experiments (Table 4). If the data of the low- and high-dose experiments are pooled and assumed to have the same infectious period distribution (scenario B), the estimated reproduction number lies between the above extremes (1.6; 95%CI: 0.90–2.5). Alternatively, if the data are pooled but the infectious period distributions are allowed to vary between the low- and high-dose experiments, the (infection-type specific) estimated reproduction numbers are 1.8 (95%CI: 1.1–3.0) and 1.2 (95%CI: 0.71–2.0) in the low- and high-dose experiments, respectively.

### Simulated epidemics

To explore the implications of the parameter estimates for the dynamics of H5N1 avian influenza in large populations of poultry we have performed stochastic simulations of an SEIR model using the parameter estimates presented in Table 4. The parameters determining the latent and infectious periods can directly be plugged into the model, but some care should be taken with the transmission parameter as it is not obvious how the parameter determining transmission between two individuals should be extrapolated to larger populations. The two common assumptions are that each individual makes a fixed number of contacts per unit of time regardless of population size (the frequency dependent transmission assumption), or that each individual makes a fixed number of contacts *with each of the other individuals* in the population per unit of time (the density dependent transmission assumption) [24]–[25]. Under the frequency dependent transmission assumption the total number of contacts that an individual makes per unit of time does not depend on total population size, while under the density dependent transmission assumption the number of contacts that an individual makes per unit of time increases linearly with total population size [24]. It is plausible that for small to moderately sized populations the transmission rate increases monotonically with increasing population size and that this increase flattens off as population size becomes large (birds cannot increase their activity levels indefinitely). Here we perform simulations of populations of 10,000 birds. In the simulations we first use the transmissibility estimates presented in Table 4, which we subsequently multiply by a factor 2. This implies that in our simulations birds in a population of 10,000 are either as active as birds that are kept in pairs, or twice as active as birds in pairs.

**Figure 4:**
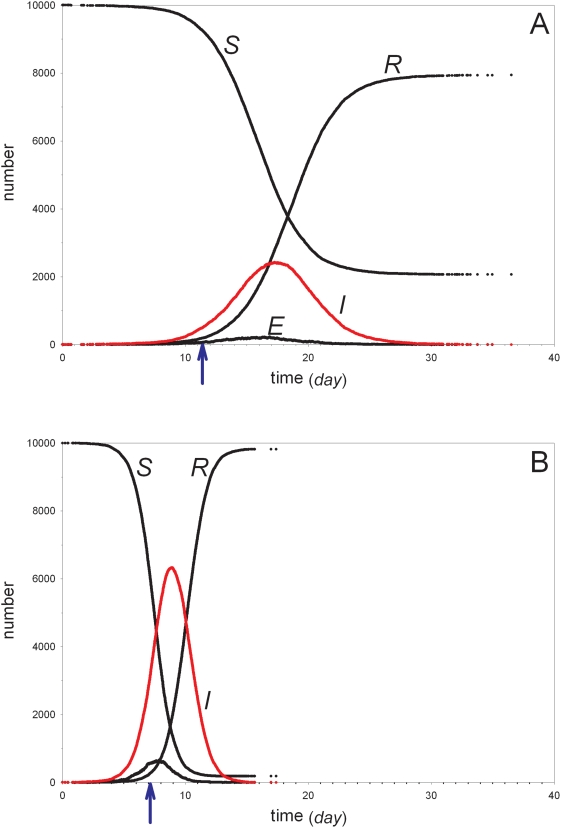
Simulations of an epidemic in a population of 50,000 birds. Parameters values are based on the estimates of Table 4 (scenario A1). A dot is plotted for the population state after each tenth event. (A) transmission parameter as in Table 4 (0.8 (*day^−1^*)) and (B) transmission parameter increased twofold (1.6 (*day^−1^*)).

Figure 4 shows two representative simulations of an epidemic in a population of 10,000 individuals using the parameter estimates of the low-dose experiment (Table 4). The top panel shows the time course of the epidemic in case of low transmissibility (leading to a reproduction number of *R = 2.0*), while the bottom panel shows the dynamics if the transmission rate parameter is increased twofold (implying a reproduction number of *R = 4.0*). The figure shows that the epidemic unfolds in about a month (top panel) to approximately two weeks (bottom panel), depending on whether the transmission parameter is small or large. Furthermore, the figure shows that the peak prevalence is about 25% of total population size if transmissibility is low, and approaches 65% if transmissibility is high. Increasing the virus' transmissibility from twofold to, say, tenfold leads to minor changes in the infection dynamics as every susceptible individual is already very quickly (within a time span of a week) infected in the high transmissibility scenario (results not shown). It is of note that in comparison with standard stochastic models that assume exponentially distributed latent and infectious periods the epidemics in Figure 4 are considerably more peaked, while their durations are substantially shorter (results not shown) [12].

Rapid detection of outbreaks of H5N1 highly pathogenic avian influenza virus in poultry is of paramount importance for efficient control within poultry flocks and to be able to minimize the opportunities of virus transmission between flocks [26]–[28]. If we assume that avian influenza can be detected with high specificity if mortality is at least 0.5% on two consecutive days [26],[29], then an outbreak will be detected in our simulations between days 11 and 12 after introduction if transmissibility is low, and between days 7 and 8 if transmissibility is high (see the blue arrows in Figure 4). In case of low transmissibility, this gives a window of opportunity of at most ten days to reduce the infectious output of the flock (Figure 4A). If, however, transmissibility is high, circulation of the virus will only be detected near the moment of peak infectivity, and there is a window of opportunity of at most five days for control measures to be effective in reducing the infectious output of infected flocks once they are detected (Figure 4B). Overall, our simulations indicate that control of H5N1 avian influenza in poultry flocks once an outbreak has been detected may be more difficult than hitherto thought [22], [26]–[27].

### Control by vaccination

To further investigate the potential for control by vaccination we have carried out simulations using estimates of the epidemiological parameters (Table 4) and efficacy of vaccination (Table S1). Because of the reasons discussed above, it is highly unlikely that an outbreak can be controlled by vaccination once it has been detected. Adding to this is the fact that it may take 7–10 days for vaccination to become effective in interfering with transmission [14]–[16]. However, it may still be possible to prevent or curb outbreaks by preventive vaccination.

**Figure 5:**
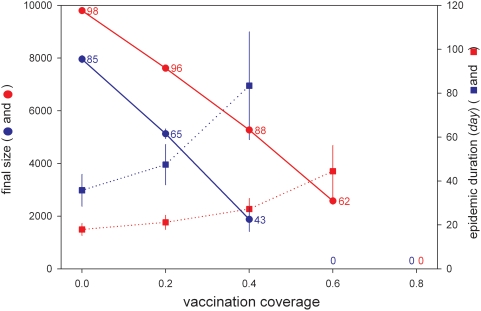
The effect of preventive vaccination as a function of vaccination coverage. Circles refer to the final size of major outbreaks (error bars: ±2SD) and squares indicate the duration of major outbreaks (error bars: ±2SD). Blue and red lines represent the low and high transmissibility scenarios, respectively (cf. Figure 4). 100 simulations are performed for each parameter constellation. Numbers near the circles refer to the number of simulations that yield a major outbreak. Major outbreaks are operationally defined as those outbreaks in which at least 50 birds are infected.

Figure 5 gives an overview of the fraction of outbreaks that yield a major outbreak (numbers near circles), the size of the major outbreaks (circles), and the duration of the epidemics (squares) as a function of the fraction of birds that is vaccinated prior to introduction of the virus. If transmissibility is low (cf. Figure 4A)(blue lines), the probability of a major outbreak as well as the size of the major outbreaks decrease with increasing vaccination coverage. The duration of major outbreaks, however, increases with increasing vaccination coverage [11]–[12]. Major outbreaks cannot occur for the parameters presented in Table 4 if coverage is at least 60%. If, on the other hand, pathogen transmissibility is high (cf. Figure 4B)(red lines), then the probability of a major epidemic and final size of the epidemics increase in comparison with the low-transmissibility scenario, while the duration of the epidemics decreases [11]. Still, both the probability of a major outbreak as well as the size of the outbreak decrease with increasing vaccination coverage, and major outbreak cannot occur if vaccination coverage is at least 80%. Summarizing, our simulations indicate that it is possible to attain a state of herd immunity by incompletely vaccinating flocks of chickens even if birds are assumed to make twice as many contacts per unit of time as estimated in our transmission experiments.

## Discussion

In this study we have attempted to fill the remarkable void of quantitative information on key epidemiological parameters of H5N1 highly pathogenic avian influenza in chickens. Our results indicate that H5N1 virus induces a short period of latency and a short infectious period. In fact, our estimate of the mean of the latent period varies from 0.20 days (95%CI: 0.049–0.43 days) in scenario A1 to 0.44 days (95%CI: 0.14–0.87 days) in scenario A2 (Table 4). Likewise, the mean infectious period varies from 1.3 days (95%CI: 0.92–1.8 days) in scenario A2 to 2.5 days (95%CI: 2.2–2.8 days) in scenario A1. Estimates of the variance of the infectious period are generally low, much lower than the corresponding means (Table 4). This implies that the distributions of the infectious periods are fairly narrow. Similar results were reported by Carrat and colleagues [30] who found that shedding of human influenza viruses increased sharply 0.5–1 day after infection, while the infectious period was centered narrowly around five days.

Our estimates of the transmission parameter are remarkably similar across the different datasets and model scenarios. The estimate of the transmission parameter is lowest if the data of all experiments are combined (median: 0.73 per day; 95%CI: 0.43–1.2 per day) and highest if the analysis allows for differences between inoculated and contact birds (median: 0.81 per day; 95%CI: 0.44–1.3 per day). In combination with the estimates of the mean infectious period these estimates yield estimates of the reproduction number varying from 0.99 (95%CI: 0.38–2.1) in the high-dose experiment (scenario A2) to 2.0 (95%CI: 0.96–3.6) in the low-dose experiment (scenario A1).

In view of the generally held belief that highly pathogenic avian influenza viruses spread easily and rapidly among chickens [14]–[16], [22], [26]–[27] our estimates of the reproduction number may seem low. In this respect a number of points are worth of discussion. First, we have assumed frequency dependent transmission, which assumes that each bird makes a fixed number of contacts per unit of time, regardless of the size of the population [24]. This is convenient since it allows one to directly extrapolate from small to large populations. The reason is that under this assumption the reproduction number does not depend on total population size. There is, moreover, evidence that a frequency dependent transmission model provides a better description of the pathogen dynamics than a density dependent model in farm animals that are generally held at a constant stocking density [31]. Still, some uncertainty remains as to how our estimates of the transmission parameter and infectious period should be combined into an estimate of the reproduction number. To address this potential problem we have in our simulations included a high transmissibility scenario (Figures 4 and 5) that in essence assumes that birds in large populations are twice as active as birds in our transmission experiments with pairs of birds.

Second, it is not straightforward to extrapolate our results that were obtained in an experimental setting to the situation in the field. This is especially so for estimates of the transmission parameter, which are the result not only of an autonomous process of viral replication and interaction of the pathogen with the immune system within a single host, but also of an interaction between different individuals. Ambient temperature, stocking density, feeding status of the birds, etcetera could all impact on this interaction and critically affect estimates of the transmission parameter. To counter this we have tried to match the conditions in our experiments to those in commercial laying chicken farms. Reassuringly, a recent analysis of transmission of H5N1 in the field [7] also indicates that the reproduction number of H5N1 virus among chickens is fairly low, ranging from 2.0 to 3.5. This suggests that our estimates of the reproduction number obtained using pairs of birds are low but not unreasonable.

A third point that deserves attention is the fact that housing systems of layer flocks vary from floor systems in which birds can mingle freely to caged systems in which no direct contact between (groups of) birds is possible. In principle, our study is aimed at quantifying transmission in a situation where there is direct contact between birds, corresponding to a floor system. However, the lone study that focused on within-flock transmission (mostly backyard flocks) did not find differences between different housing systems, suggesting that if there are differences in the transmission dynamics they cannot be large [7]. Nevertheless, more information on the infection dynamics in the field would be highly welcome to help bridging the gap between findings obtained in experimental studies and the situation in the field.

While it is not straightforward to extrapolate from our experimental setting to the field situation, experimental transmission studies also have distinct advantages over field studies. In particular, while field studies often suffer from various sources of bias and confounding, this is not the case in an experimental setting. This allows one to directly ascribe differences between control and treatment groups directly to the treatment (e.g., vaccination) since all other animal and environmental conditions are held constant. Moreover, an experiment has the added advantage over a field study that far fewer birds are needed and that the birds can be sampled more often and efficiently than in a field study. This has allowed us to obtain precise estimates of the key epidemiological parameters of H5N1 highly pathogenic avian influenza in unvaccinated chickens using no more than 50 birds.

Our results show remarkable differences between experiments in which the inoculated bird received a low infection dose (0.2*10^5^ EID_50_) and experiments in which the inoculated bird received a high dose (0.2*10^6^ EID_50_). Specifically, while 9 out of 11 birds were infected in case of a low infection dose (Table 1), only 5 out of 11 were infected in case of a high infection dose (Table 2). This is an interesting and counterintuitive result, which is likely to result from the fact that the infectious period in the experiments in which the inoculated bird received a high inoculation dose is significantly smaller than in the experiments in which the inoculation dose was low or in which the infectious period of the naturally infected birds was estimated separately (low dose: mean 2.5 days (95%CI: 2.2–2.8); high dose: mean 1.3 days (95%CI: 0.92–1.8); contact birds only: mean 2.5 days (95%CI: 1.9–3.3)). Earlier experimental transmission studies with H7N7 highly pathogenic avian influenza virus (A/Chicken/Netherlands/621557/03) in a variety of birds and H5N1 highly pathogenic avian influenza virus (A/Chicken/China/1204/04, also designated A/Chicken/GxLA/1204/04) in ducks used an infection dose of 0.2*10^6^ EID_50_ since this yielded comparable infections in inoculated and naturally infected animals [14]–[16]. The finding that the infection dose is of importance in determining the duration of infection is of both theoretical and practical relevance as it suggests that the infection pressure in the population may not only determine the incidence of infection but also the course of infection. If it is typical that a low infection dose is associated with a long infectious period while a high infection dose generally leads to infections that are of short duration, then this would necessitate a rethinking of the critical determinants of H5N1 avian influenza transmission in populations of birds, and it could potentially have profound implications for optimal control and containment strategies.

To investigate the implications of our parameter estimates for the dynamics of H5N1 avian influenza virus in large groups of chickens we have carried out stochastic simulations. Since it is not obvious how the transmission parameter as estimated between pairs of chickens can be extrapolated to large populations, we considered a low and high transmissibility scenario (Figure 4). The simulations indicate that, even if we assume that the transmission parameter is small, the epidemic usually unfolds in about a month, and that once the epidemic has taken off it only takes about two weeks to come to an end. If, as appears more likely, the transmission rate is larger in large population than in populations of two birds, then the epidemic takes off more quickly after a primary introduction, and also comes to an end more quickly. For control purposes this implies that it will be very difficult, if not impossible, to effectively control an outbreak once it has been detected. It may even prove difficult to reduce transmission opportunities from an infected population (a farm, say) to susceptible populations, as the number of dead birds may start to rise just before peak infectivity (Figure 4). This suggests that perhaps other indicators of infection, such as lethargy, reduced feed or water intake should be added to the mortality indicator to obtain a sensitive syndrome-reporting system [32].

While H5N1 virus spreads rapidly among unvaccinated chickens, no transmission was observed at all in the experiments with inactivated oil emulsion vaccines (Tables S1, S2, S3, S4, S5, S6, and S7). This was true not only for an H5N1 vaccine virus which had 100% homology to the challenge virus, but also for genetically distant heterologous viruses that contained inactivated H5N2 viruses. These findings indicate that it is possible, at least in principle, to reduce transmission by vaccination to the extent that no epidemics can occur. This suggestion is corroborated by our simulations which indicate that a vaccination coverage as low as 60%–80% may already be sufficient to obtain herd immunity (Figure 5). Of course, it should be borne in mind that in our experiments all birds received two vaccination doses, that the timing of challenge (two weeks after the last vaccination bout) was probably ideal, and that in the field there are various factors that may interfere with vaccination (concurrent infections, immune depression by various causes) [33]. Still, our results and those of others [34]–[36] provide a proof-of-principle that herd immunity can be obtained with currently available inactivated oil emulsion vaccines. The finding that H5N1 avian influenza virus has a lower transmissibility than hitherto believed [26] also implies that outbreaks may be easier to prevent than previously thought, since the reproduction number is already relatively close to the threshold value of 1.

## Methods

### Birds and housing

All experiments were carried out in PT Medion laboratories in Bandung, Indonesia, which have high containment facilities (BSL3). In all experiments, specific pathogen-free (SPF) layer chickens from the animal unit of Medion were used. The birds were hatched and housed in one group until 4 weeks of age. At that age, pairs of birds were housed in cages. Three rooms were available to house the various vaccinated and unvaccinated pairs of birds. Two rows with three levels of cages on top of each other were available in each room. The rows with cages were separated by a corridor of approximately 1 m width. The various rooms as well as the rows with the cages had separate ventilation systems. Each cage had a separate feeding and drinking system. The floor and walls of each cage were covered with plastic to prevent spread of manure or other materials between cages. When sampling the birds, animal caretakers used a new pair of gloves for each cage. Unvaccinated sentinel birds were placed at regular distances between the cages used in the experiments to ensure that no transmission had taken place between cages. All sentinels survived and remained seronegative during the course of the experiments.

### Virus, vaccines and inoculum

The challenge strain used in the experiments was A/Chicken/Legok/2003 H5N1, a highly pathogenic H5N1 strain isolated in Indonesia in 2003 which is genetically very close to strains that circulate in Indonesia in 2008. The strain has been used in experiments carried out at Medion and is able to induce infection, typical signs of disease, and high mortality rates in chickens.

Inactivated oil emulsion vaccines were available from three different manufactures: PT Medion (Bandung, Indonesia), PT Vaksindo (Bogor, Indonesia) and Intervet (Mexico). The vaccines contained either an H5N1 or H5N2 virus strain. The H5N1 vaccines contained A/Chicken/Legok/2003 H5N1, i.e. the vaccine and challenge strains were identical. The H5N2 vaccines contained either A/Turkey/England/N28/73 H5N2 or A/Chicken/Mexico/232/94/CPA H5N2. The protein homologies of the antigenic part of the hemagglutinin (HA1) of the challenge strain to the H5N2 A/Turkey/England/N28/73 and H5N2 A/Chicken/Mexico/232/94/CPA vaccine strains are 92% and 86%, respectively.

All vaccines were re-vialed in coded bottles, and the identity of the vaccines was not known to the staff involved in the experiment. In this manner the experiments were double blinded.

Because the size of a natural infection dose is unknown the inoculum consisted of diluted allantoic fluid containing either 10^5^ EID_50_ per ml (low inoculation dose) or 10^6^ EID_50_ per ml (high inoculation dose). The birds were inoculated both intranasally (0.1 ml) and intratracheally (0.1 ml). Virus titres were confirmed before and after inoculation by titration on embryonated SPF eggs.

### Experimental design

Each experiment consisted of a set of 11 trials. In each of the trials an inoculated bird was placed in a cage with an uninfected contact bird, and the transmission chain was monitored daily by virus isolation performed on swabs taken from the trachea and cloaca. In all, a total of eight experiments were carried out. Unvaccinated birds were used in two experiments. In the first of these the inoculated birds received a low infection dose, while in the second the inoculated birds received a high infection dose. The remaining six experiments with vaccinated birds differed with respect to the vaccine used, the manufacturer, and the inoculation dose. Tables 1 and 2 show the data of experiments with unvaccinated birds, and Tables S1, S2, S3, S4, S5, S6, and S7 give an overview of the experiments with vaccinated birds.

At 4 weeks of age all birds of the vaccination experiments received their first vaccination dose. A second vaccination was carried out at 7 weeks of age. At 10 weeks of age (day 0) one bird was chosen at random per cage, taken from the cage, and infected intratracheally and intranasally. To avoid direct infection of the contact bird by the inoculum the artificially infected birds were placed back in their cages only after a delay of 8 hours.

### Sampling and testing

Tracheal and cloacal swabs were taken daily for 10 days after challenge from all birds. Swabs were incubated for 1 h in one ml of PBS medium containing antibiotics. The medium was subsequently stored at −70°C until testing. Three embryonated SPF chicken eggs were injected with 0.2 ml of the swab medium per egg. After culture for 4 days or when embryos died, the allantoic fluid was harvested and a hemagglutination (HA) assay was performed following standard procedures (www.oie.int). When at least one of the eggs was positive in the hemagglutination assay the swab was considered to be positive.

The serological status of the birds was determined just before vaccination, at the start of the experiments just before inoculation (day 0) and, for birds that survived, at the end of the experiments (day 14). Serum blood samples were taken from all birds by puncturing the wing vein. Blood samples were centrifuged and serum was stored at −20°C until tested. The sera were tested in the hemagglutination inhibition (HI) test according procedures described in the Manual of Diagnostic Tests and Vaccines for Terrestrial Animals of the OIE (www.oie.int) using 4 HA units (HAU) of A/Chicken/Legok/03 H5N1 as antigen. Titres were expressed as ^2^log of the serum dilution that caused complete inhibition of agglutination, as specified by OIE guidelines.

Clinical signs of disease were recorded daily for a period of up to 10 days after challenge.

### Statistical analysis

As a first step we estimated the reproduction number *R* by final size methods [14]–[17]. Since each trial contains only one inoculated bird and one susceptible contact bird, the likelihood function takes the following simple form:
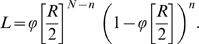
(1)In this equation *N* and *n* are the number of trials per experiment and the number of infected contact birds, while 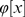 represents the Laplace transform of the infectious period probability distribution when the mean infectious period is scaled to 1. Hence, 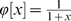 in case of an exponentially distributed infectious period, and 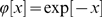 in case of a fixed infectious period. Table 3 provides estimates of the reproduction number with corresponding 95% confidence intervals, as well as p-values of the null-hypothesis that the reproduction number is greater than or equal to the threshold value of 1 [23].

In a second step, we estimated all parameters of interest by Bayesian methods [18]–[21]. In the following we denote by 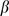 the transmission rate parameter, by 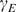 and 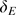 the parameters determining the latent period probability distribution, and by 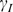 and 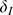 the parameters of the infectious period probability distribution. We assume that the latent and infectious periods are gamma distributed, and that 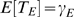 and 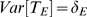, and 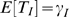 and 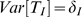 represent the means and variances of these distributions. The corresponding probability densities are denoted by 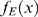 and 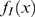.

Further, 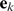, 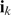, and 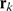 are *N*-dimensional vectors which contain the time points of the S→E, E→I, and I→R transitions for inoculated (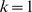) and contact (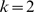) birds in the *N* trials. Hence, we have 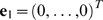 by definition, while all other transition times are unknown. The unknown transitions are added in the analyses by Bayesian imputation. We adopt the convention that 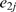 denotes the exact time at which the contact bird in experiment *j* is infected, that 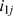 denotes the exact time that the inoculated bird in experiment *j* became infectious, etcetera.

With these notational conventions, the contribution of trial *j* to the likelihood is given by
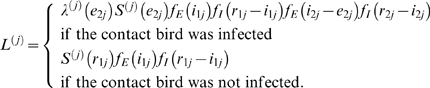
(2)In the above equation 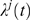 and 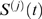 denote the infection hazard in trial *j* at time *t* and the probability that the contact bird in trial *j* remains uninfected up to time *t*, respectively. If we let […] denote the indicator function, the infection hazard is given by
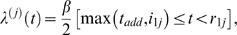
(3)where the parameter 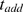 represents the delay between the moment of inoculation and the placing back of the inoculated birds in their cages, and the function 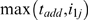 marks the beginning of the at-risk period for the contact bird. In all trials and experiments, the delay is 8 hours, i.e. 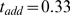 (*day*). The probability that the contact bird in trial *j* remains uninfected up to time *t* can be expressed in terms of the infection hazard as follows
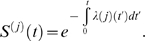
(4)Using Equations (2)–(4) the likelihood function is given by the product of the contributions of the individual trials:
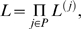
(5)where *P* represents the set of trials. Equations (2)–(5) form the basis of the analyses in Figures 1, S1, S2, and S3.

The likelihood contribution in Equation (2) assumes that the latent and infectious periods of inoculated and infectious birds are identically distributed. To investigate the validity of these assumptions we also considered a model which allows for differences between the inoculated and contact birds. In this case, the likelihood contribution becomes
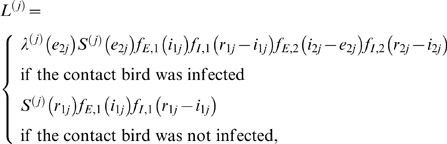
(6)where 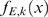 and 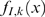 are the probability density functions of the latent and infectious periods of the inoculated birds (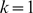) and contact birds (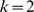). The results of the analyses based on Equation (6) are given in Figure 3 and Figure S5. In a similar manner, the likelihood contribution in Equation (2) is adapted to allow for differences in the infectious period in the low- versus high-dose experiments. The results of these analyses are summarized in Figure 2 and Figure S4.

Notice that, since the transmission rate in Equations (1), (2), and (5) is divided by the total size of the population (i.e. 2), the above model assumes frequency dependent transmission (as opposed to density dependent transmission) [24]. For the present experimental setup with one inoculated bird and one contact bird, the value of the transmission parameter of the density dependent model is simply given by the transmission rate parameter of the frequency dependent transmission model divided by 2 (the size of the population). In case of a frequency dependent transmission model the (basic) reproduction number is given by the product of the transmission rate parameter and the mean infectious period: 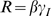. In case of a density dependent transmission model the reproduction number is a function of population size, and it is given by 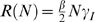, where 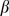 denotes the transmission parameter of the frequency dependent model with two birds [25].

As in earlier papers [18]–[21] the epidemiological parameters of interest (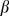, 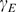, 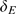, 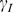, and 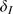) were estimated by Bayesian methods of inference using Markov Chain Monte Carlo. Throughout, all prior distributions of the parameters were uniformly distributed on the interval (0.001–5). As an alternative we also considered vague gamma prior distributions, and obtained comparable results (results not shown).

In our simulations the epidemiological parameters and unobserved transitions were updated by a random-walk Metropolis algorithm. We used Normal proposal distributions with the current value as mean, and a standard deviation of 0.025, 0.05, or 0.1. The transmission parameters and unobserved transitions were updated in blocks, in the order 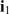, 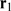, 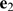, 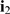, 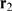, and 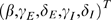. Notice that updating of the individual transition vectors needs to take into account the infection data of Tables 1 and 2 and the information contained in the other transition vectors, as these specify the admissible intervals of the various transitions. The above updating scheme yielded chains that converged quickly and showed satisfactory mixing. In all analyses we took a burn-in of 25,000 cycles and a simulation length of 200,000 cycles. Thinning was applied by taking only each 100th cycle as a sample from the posterior distribution. We performed four replicate simulations to check the precision of the parameter estimates obtained by the above procedures. These simulations yielded parameter estimates and 95% credible intervals that were close to those given in Table 4.

To choose between models of different complexity we made use of Bayes factors (BF) [37]. To this end the marginal likelihoods of competing models were estimated by importance sampling using the harmonic means of the posterior likelihood values [37]. The BF converged slowly, possibly because of the high dimensionality of the model (86 unobserved transition events plus 5–9 epidemiological parameters), the mutual dependencies of the unobserved transitions, and the fact that the likelihood is strongly affected by the parameters defining the variances of the latent and infectious periods if those are small. However, this did not appear to be a major practical problem as differences between competing models were usually large. When reported in the text we calculated the BF of the pair of simulations that had the smallest difference in marginal likelihoods.

A suite of Bayesian analyses were performed for the experiments with unvaccinated birds. First, we analyzed the low- and high-dose experiments of Tables 1 and 2 separately (scenarios A1 and A2). Second, we pooled the data of the low- and high-dose experiments (scenario B). We then considered an integrated analysis of the two experiments that allowed for differences in the infectious periods in the low- versus high-dose experiments (scenario C). Finally, we considered a scenario which allowed for differences in the epidemiological characteristics of the inoculated and contact birds (scenario D).

### Simulated epidemics

To explore the implications of the parameters estimated by the above procedures for the pathogen dynamics in large groups of birds, we performed simulations of the stochastic SEIR model using the Sellke construction [16]. In the simulations we assumed gamma distributed latent and infectious periods, and used the medians of the parameter estimates of Table 4 as input values. The programs for the MCMC analyses and simulated epidemics were written in Mathematica 6.0 (www.wolfram.com).

## Supporting Information

Figure S1 Bayesian analysis of the low-dose experiment (Table 1). Shown are samples of the mean of the latent period (A), variance of the latent period (B), mean of the infectious period (C), variance of the infectious period (D), transmission rate parameter (E), mean of the infectious period versus transmission rate parameter (F), mean versus variance of the latent period (G), and mean versus variance of the infectious period (H).
(4.87 MB TIF)

Figure S2 Bayesian analysis of the high-dose experiment (Table 2). Shown are samples of the mean of the latent period (A), variance of the latent period (B), mean of the infectious period (C), variance of the infectious period (D), transmission rate parameter (E), mean of the infectious period versus transmission rate parameter (F), mean versus variance of the latent period (G), and mean versus variance of the infectious period (H).
(4.80 MB TIF)

Figure S3 Bayesian analysis of the combined experiments (scenario B). Shown are samples of the mean of the latent period (A), variance of the latent period (B), mean of the infectious period (C), variance of the infectious period (D), transmission rate parameter (E), mean of the infectious period versus transmission rate parameter (F), mean versus variance of the latent period (G), and mean versus variance of the infectious period (H).
(4.83 MB TIF)

Figure S4 Bayesian analysis of the combined experiments (scenario C). Shown are samples of the mean of the latent period (A), variance of the latent period (B), mean of the infectious period (C), variance of the infectious period (D), transmission rate parameter (E), mean of the infectious period versus transmission rate parameter (F), mean versus variance of the latent period (G), and mean versus variance of the infectious period (H). Red and blue dots refer to parameters characterizing the low- and high-dose experiments, respectively.
(5.48 MB TIF)

Figure S5 Bayesian analysis of the combined experiments (scenario D). Shown are samples of the mean of the latent period (A), variance of the latent period (B), mean of the infectious period (C), variance of the infectious period (D), transmission rate parameter (E), mean of the infectious period versus transmission rate parameter (F), mean versus variance of the latent period (G), and mean versus variance of the infectious period (H). Blue and red dots refer to parameters characterizing the inoculated and contact birds, respectively.
(6.38 MB TIF)

Table S1 Overview of the experiments with vaccinated birds.
(0.03 MB DOC)

Table S2 Overview of experiment #1 (see Table S1) with vaccinated birds inoculated with a low virus dose. The vaccine contained a heterologous H5N2 vaccine strain (A/Turkey/England/N28/73).
(0.06 MB DOC)

Table S3 Overview of experiment #2 (see Table S1) with vaccinated birds inoculated with a low virus dose. The vaccine contained a heterologous H5N2 vaccine strain (A/Turkey/England/N28/73), from a different producer than in Table S2.
(0.05 MB DOC)

Table S4 Overview of experiment #3 (see Table S1) with vaccinated birds inoculated with a low virus dose. The vaccine contained a heterologous H5N2 vaccine strain (A/Chicken/Mexico/232/94/CPA).
(0.06 MB DOC)

Table S5 Overview of experiment #4 (see Table S1) with vaccinated birds inoculated with a high virus dose. The vaccine contained a homologous H5N1 vaccine strain (A/Chicken/Legok/2003).
(0.06 MB DOC)

Table S6 Overview of experiment #5 (see Table S1) with vaccinated birds inoculated with a high virus dose. The vaccine contained a homologous H5N1 vaccine strain (A/Chicken/Legok/2003), from a different producer than in Table S5.
(0.06 MB DOC)

Table S7 Overview of experiment #6 (see Table S1) with vaccinated birds inoculated with a high virus dose. The vaccine contained a heterologous H5N2 vaccine strain (A/Chicken/Mexico/232/94/CPA).
(0.06 MB DOC)
