# Supplementary figures and images for: Estimation of Transmission Parameters of H5N1 Avian Influenza Virus in Chickens
Source: PLoS Pathog. 2009 Jan 30;5(1):e1000281. doi: 10.1371/journal.ppat.1000281 (PMC2627927; doi:10.1371/journal.ppat.1000281)

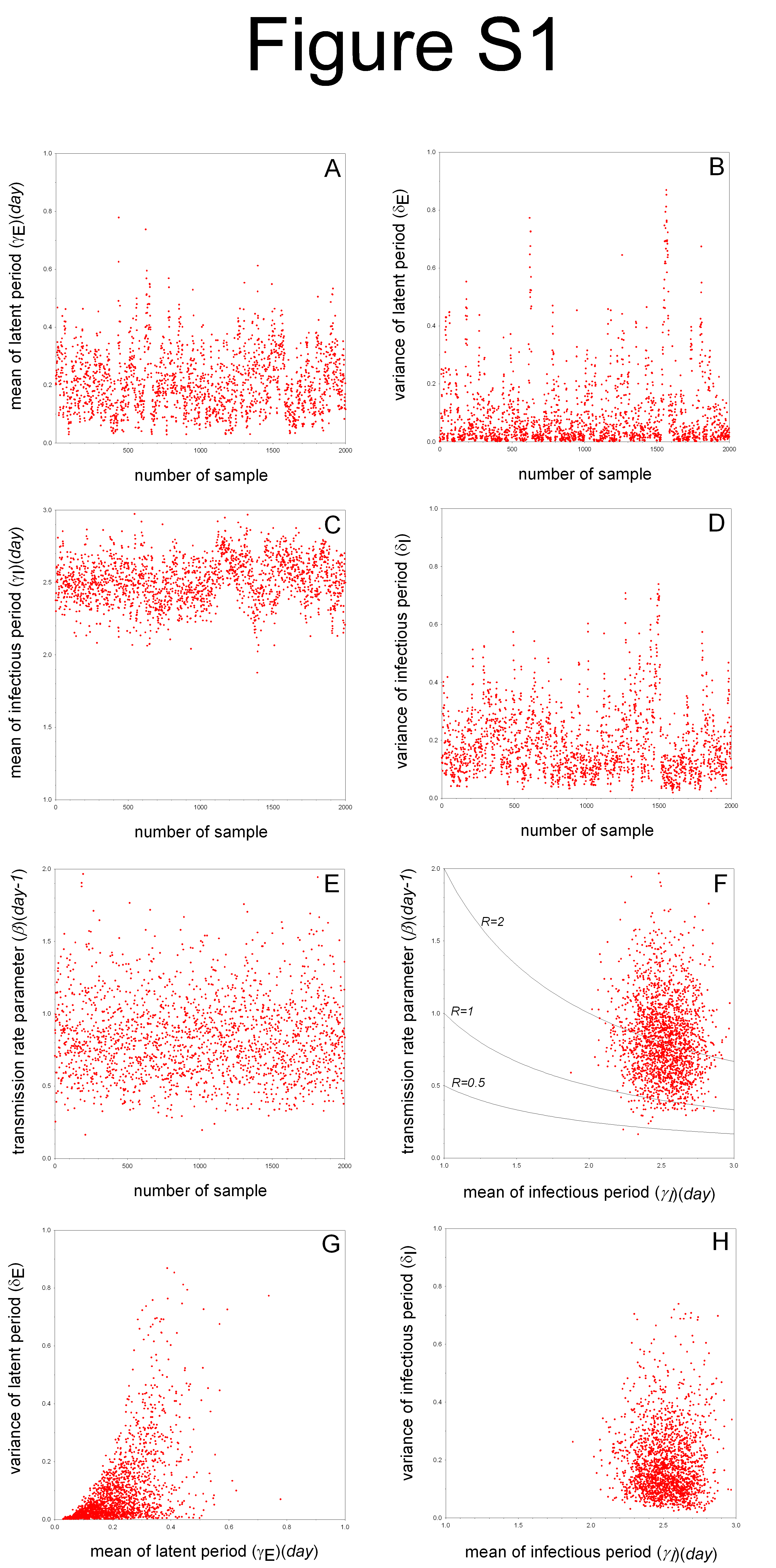

Supplement: Figure S1 — Bayesian analysis of the low-dose experiment (Table 1). Shown are samples of the mean of the latent period (A), variance of the latent period (B), mean of the infectious period (C), variance of the infectious period (D), transmission rate parameter (E), mean of the infectious period versus transmission rate parameter (F), mean versus variance of the latent period (G), and mean versus variance of the infectious period (H). (4.87 MB TIF) [file ppat.1000281.s001.tif]

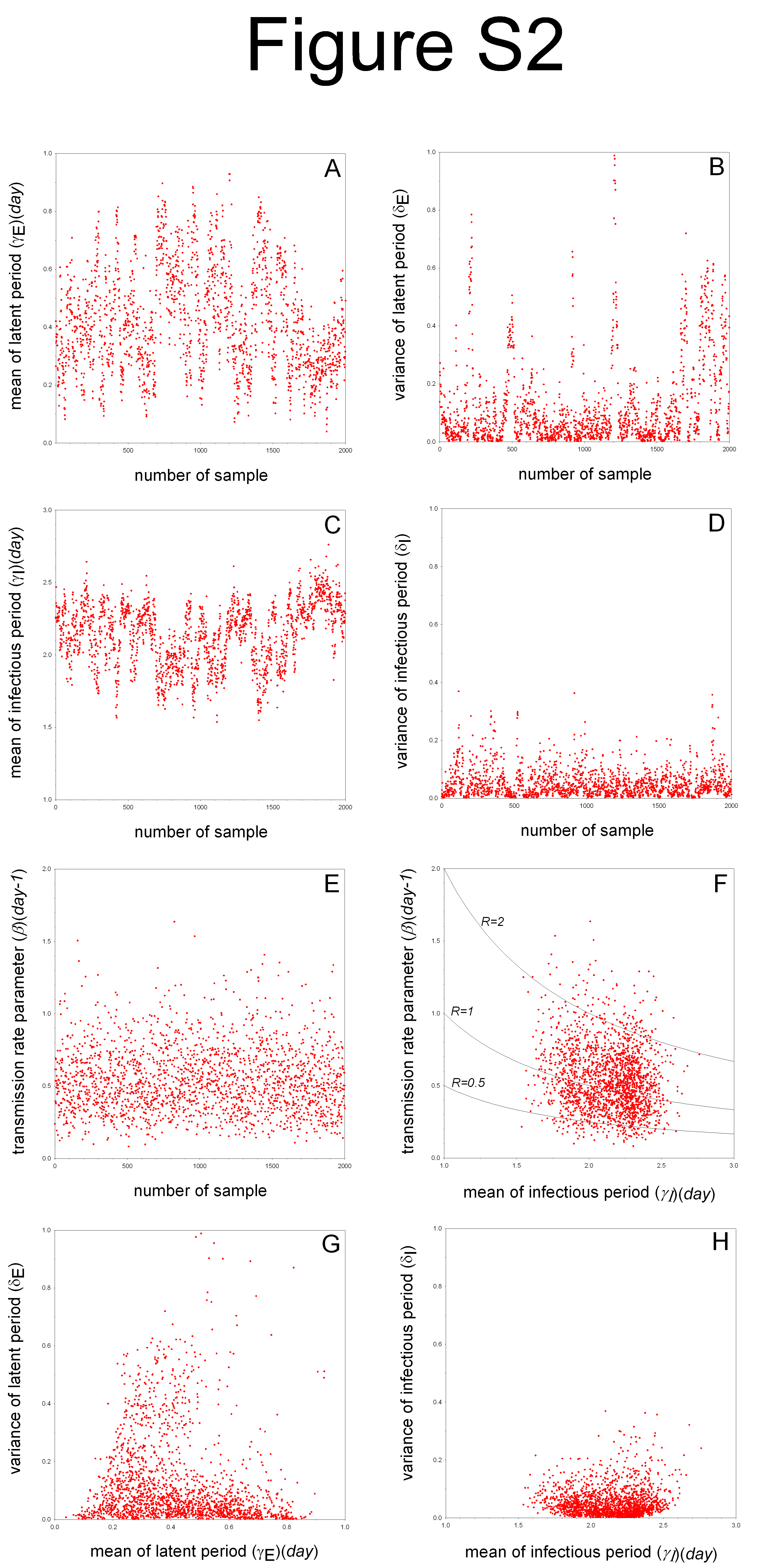

Supplement: Figure S2 — Bayesian analysis of the high-dose experiment (Table 2). Shown are samples of the mean of the latent period (A), variance of the latent period (B), mean of the infectious period (C), variance of the infectious period (D), transmission rate parameter (E), mean of the infectious period versus transmission rate parameter (F), mean versus variance of the latent period (G), and mean versus variance of the infectious period (H). (4.80 MB TIF) [file ppat.1000281.s002.tif]

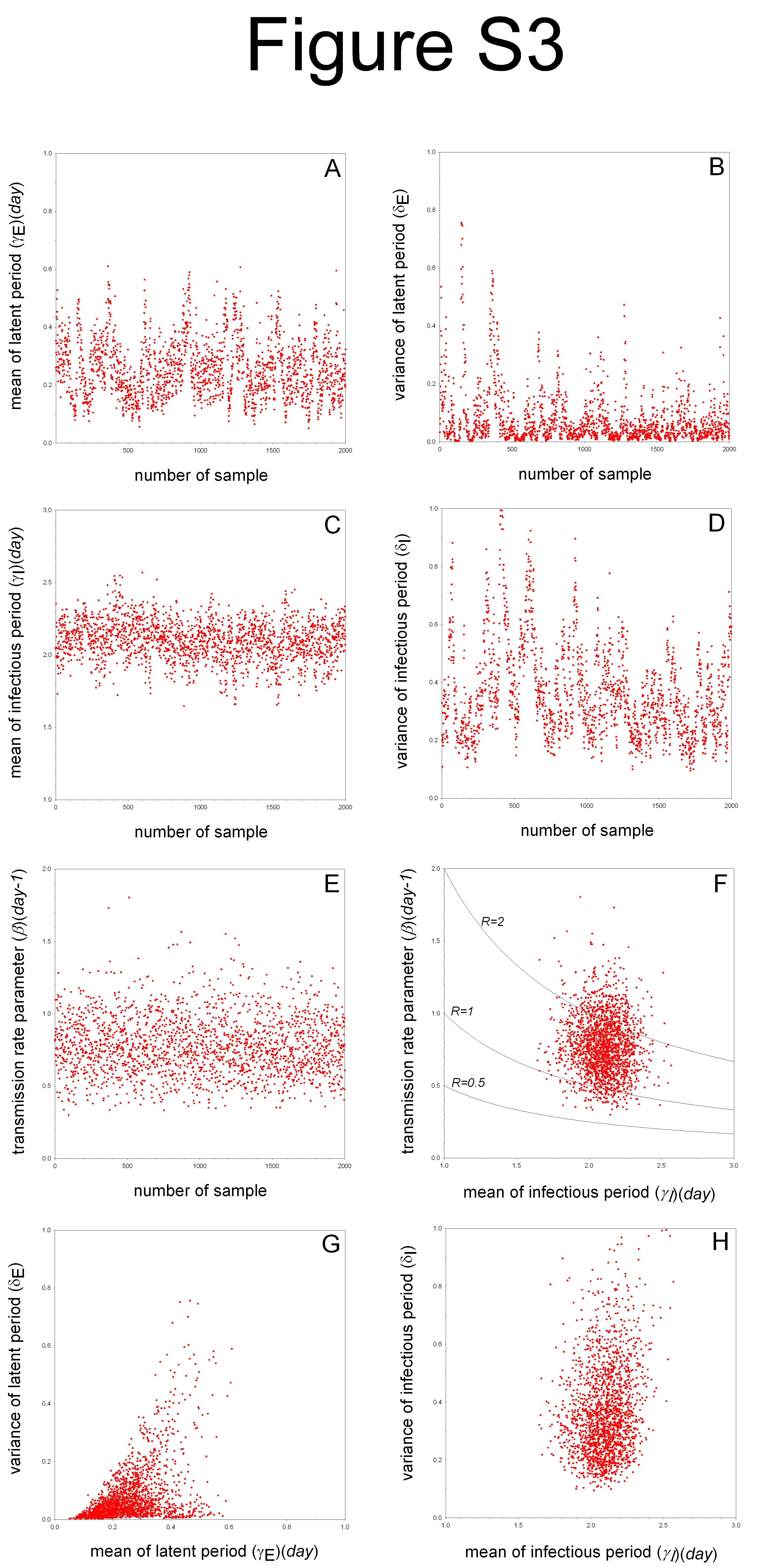

Supplement: Figure S3 — Bayesian analysis of the combined experiments (scenario B). Shown are samples of the mean of the latent period (A), variance of the latent period (B), mean of the infectious period (C), variance of the infectious period (D), transmission rate parameter (E), mean of the infectious period versus transmission rate parameter (F), mean versus variance of the latent period (G), and mean versus variance of the infectious period (H). (4.83 MB TIF) [file ppat.1000281.s003.tif]

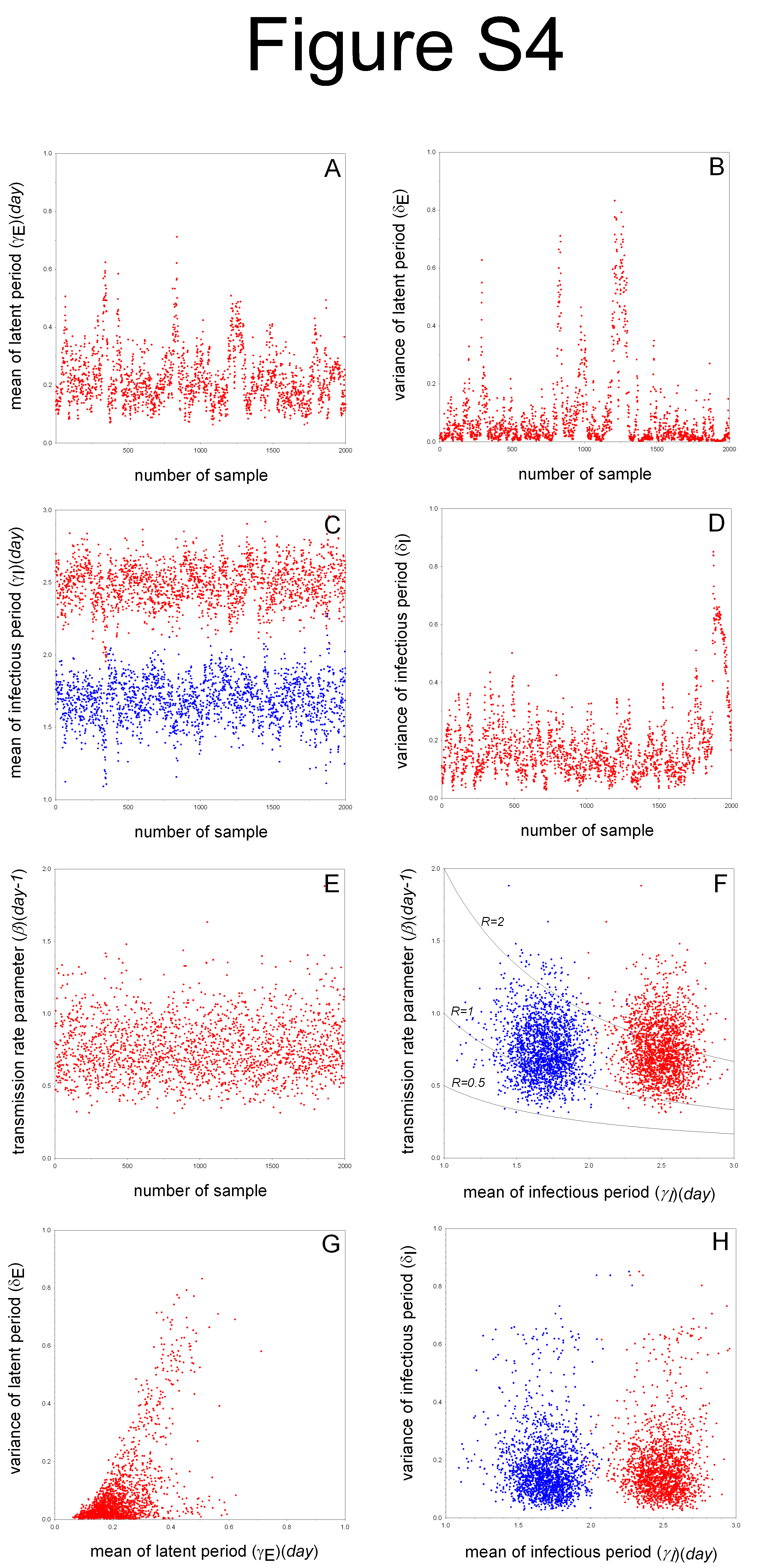

Supplement: Figure S4 — Bayesian analysis of the combined experiments (scenario C). Shown are samples of the mean of the latent period (A), variance of the latent period (B), mean of the infectious period (C), variance of the infectious period (D), transmission rate parameter (E), mean of the infectious period versus transmission rate parameter (F), mean versus variance of the latent period (G), and mean versus variance of the infectious period (H). Red and blue dots refer to parameters characterizing the low- and high-dose experiments, respectively. (5.48 MB TIF) [file ppat.1000281.s004.tif]

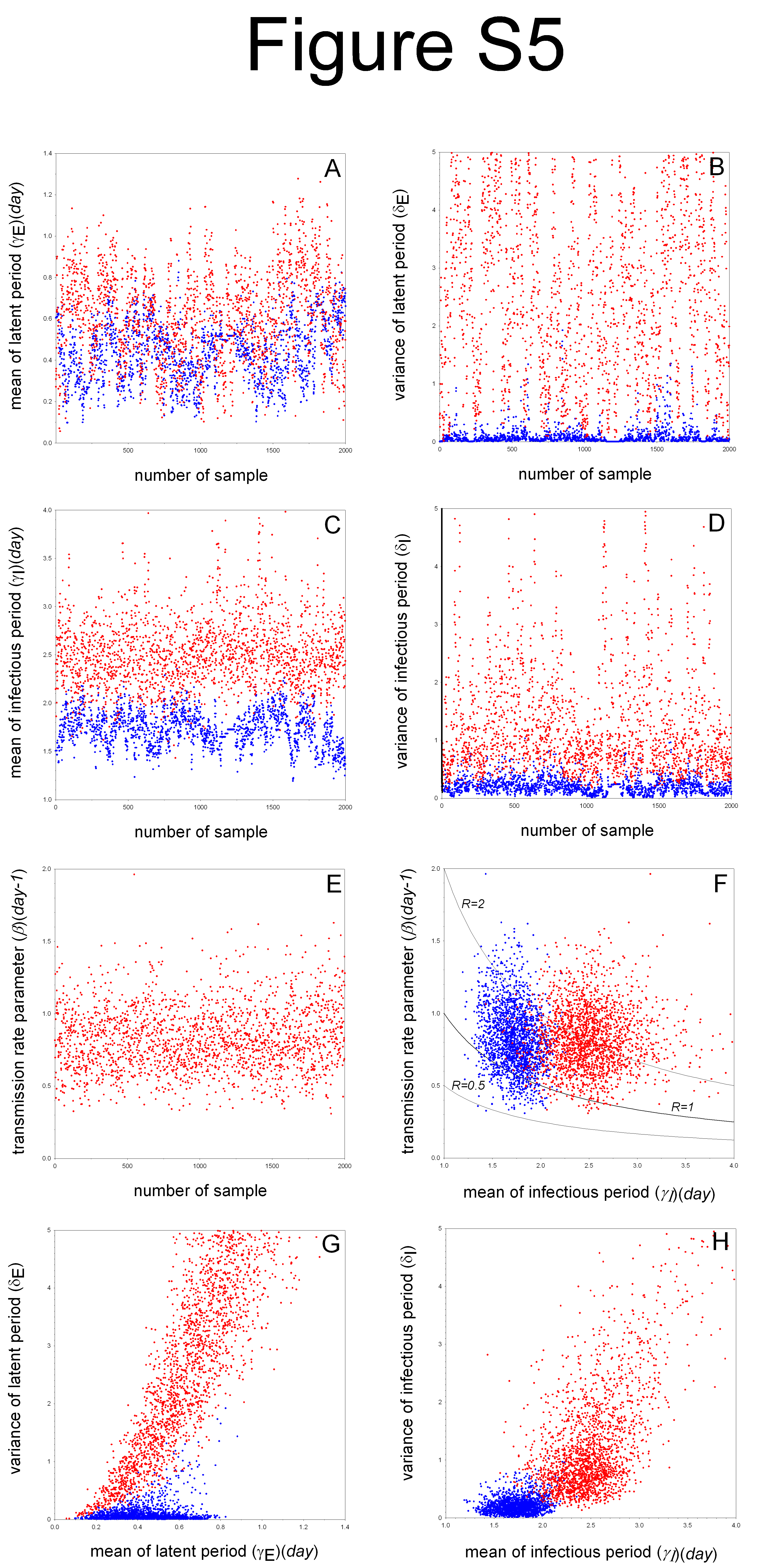

Supplement: Figure S5 — Bayesian analysis of the combined experiments (scenario D). Shown are samples of the mean of the latent period (A), variance of the latent period (B), mean of the infectious period (C), variance of the infectious period (D), transmission rate parameter (E), mean of the infectious period versus transmission rate parameter (F), mean versus variance of the latent period (G), and mean versus variance of the infectious period (H). Blue and red dots refer to parameters characterizing the inoculated and contact birds, respectively. (6.38 MB TIF) [file ppat.1000281.s005.tif]
