# Supplementary material for: Estimation of Transmission Parameters of H5N1 Avian Influenza Virus in Chickens
Source: PLoS Pathog. 2009 Jan 30;5(1):e1000281. doi: 10.1371/journal.ppat.1000281 (PMC2627927; doi:10.1371/journal.ppat.1000281)
Supplement: Table S1 — Overview of the experiments with vaccinated birds. (0.03 MB DOC) [file ppat.1000281.s006.doc]

Table S1. Overview of the experiments with vaccinated birds.

| ID | vaccine strain | inoculation dose^*^ | virus-vaccine homology^#^ | final size  (infected/total) |
| --- | --- | --- | --- | --- |
| 1 | H5N2  A/Turkey/England/N28/73 | low | 92% | 0/11 |
| 2 | H5N2  A/Turkey/England/N28/73 | low | 92% | 0/11 |
| 3 | H5N2 A/Chicken/Mexico/232/94/CPA | low | 86% | 0/11 |
| 4 | H5N1  A/Chicken/Legok/2003 | high | 100% | 0/11 |
| 5 | H5N1  A/Chicken/Legok/2003 | high | 100% | 0/11 |
| 6 | H5N2 A/Chicken/Mexico/232/94/CPA | high | 86% | 0/11 |

^*^: the inoculation dose is 0.2 ml of 10^5^ EID_50_ (low inoculation dose) and 10^6^ EID_50_ (high inoculation dose and vaccination experiments) administrated intranasally (0.1 ml) and intraocularly (0.1 ml).

^#^: percentage homology between vaccine and challenge virus of HA1 at the protein level.
