# Supplementary material for: Estimation of Transmission Parameters of H5N1 Avian Influenza Virus in Chickens
Source: PLoS Pathog. 2009 Jan 30;5(1):e1000281. doi: 10.1371/journal.ppat.1000281 (PMC2627927; doi:10.1371/journal.ppat.1000281)
Supplement: Table S7 — Overview of experiment #6 (see Table S1) with vaccinated birds inoculated with a high virus dose. The vaccine contained a heterologous H5N2 vaccine strain (A/Chicken/Mexico/232/94/CPA). (0.06 MB DOC) [file ppat.1000281.s012.doc]

Table S7. Overview of experiment #6 (see Table S1) with vaccinated birds inoculated with a high virus dose. The vaccine contained a heterologous H5N2 vaccine strain (A/Chicken/Mexico/232/94/CPA).

|  | days post challenge | | | | | | | |
| --- | --- | --- | --- | --- | --- | --- | --- | --- |
| bird type | 0 | 1 | 2 | 3 | 4 | 5 | 6 | 7 |
| i | -/- | -/- | -/- | -/- | -/- | -/- | -/- | -/- |
| c | -/- | -/- | -/- | -/- | -/- | -/- | -/- | -/- |
| i | -/- | -/- | -/- | -/- | -/- | -/- | -/- | -/- |
| c | -/- | -/- | -/- | -/- | -/- | -/- | -/- | -/- |
| i | -/- | -/- | -/- | -/- | -/- | -/- | -/- | -/- |
| c | -/- | -/- | -/- | -/- | -/- | -/- | -/- | -/- |
| i | -/- | -/- | -/- | -/- | -/- | -/- | -/- | -/- |
| c | -/- | -/- | -/- | -/- | -/- | -/- | -/- | -/- |
| i | -/- | -/- | -/- | -/- | -/- | -/- | -/- | -/- |
| c | -/- | -/- | -/- | -/- | -/- | -/- | -/- | -/- |
| i | -/- | -/- | -/- | -/- | -/- | -/- | -/- | -/- |
| c | -/- | -/- | -/- | -/- | -/- | -/- | -/- | -/- |
| i | -/- | -/- | -/- | -/- | -/- | -/- | -/- | -/- |
| c | -/- | -/- | -/- | -/- | -/- | -/- | -/- | -/- |
| i | -/- | -/- | -/- | -/- | -/- | -/- | -/- | -/- |
| c | -/- | -/- | -/- | -/- | -/- | -/- | -/- | -/- |
| i | -/- | -/- | -/- | -/- | -/- | -/- | -/- | -/- |
| c | -/- | -/- | -/- | -/- | -/- | -/- | -/- | -/- |
| i | -/- | -/- | -/- | -/- | -/- | -/- | -/- | -/- |
| c | -/- | -/- | -/- | -/- | -/- | -/- | -/- | -/- |
| i | -/- | -/- | -/- | -/- | -/- | -/- | -/- | -/- |
| c | -/- | -/- | -/- | -/- | -/- | -/- | -/- | -/- |

i: inoculated bird; c: contact bird; x/y: test result for virus isolation in the trachea/cloaca.
